# Naltrexone blocks alcohol-induced effects on kappa-opioid receptors in the plasma membrane

**DOI:** 10.21203/rs.3.rs-3091960/v1

**Published:** 2023-07-21

**Authors:** Lars Terenius, Sho Oasa, Erdinc Sezgin, Yuelong Ma, David Horne, Mihajlo Radmiković, Tijana Jovanović-Talisman, Remi Martin-Fardon, Vladana Vukojevic

**Affiliations:** Karolinska Institutet; Karolinska Institutet; Karolinska Institute; The Scripps Research Institute; Karolinska Institute

## Abstract

Naltrexone (NTX), a homologue of the opiate antidote naloxone, is an orally active long-acting mu-opioid receptor (MOP) antagonist used in the treatment of opiate dependence. NTX is also found to relieve craving for alcohol and is one of the few FDA-approved drugs for alcohol use disorder (AUD). Reports that NTX blocks the actions of endogenous opioids released by alcohol are not convincing, suggesting that NTX interferes with alcohol actions by affecting opioid receptors. MOP and kappa-opioid receptor (KOP) are structurally related but functionally different. MOP is mainly located in interneurons activated by enkephalins while KOP is located in longer projections activated by dynorphins. While the actions of NTX on MOP are well established, the interaction with KOP and addiction is not well understood. We used sensitive fluorescence-based methods to study the influence of alcohol on KOP and the interaction between KOP and NTX. Here we report that alcohol interacts with KOP and its environment in the plasma membrane. These interactions are affected by NTX and are exerted both on KOP directly and on the plasma membrane (lipid) structures (“off-target”). The actions of NTX are stereospecific. Selective KOP antagonists, recently in early clinical trials for major depressive disorder, block the receptor but do not show the full action profile of NTX. The therapeutic effect of NTX treatment in AUD may be due to direct actions on KOP and the receptor environment.

## INTRODUCTION

Moderate consumption of alcohol (i.e., social drinking) gives a sensation of elatedness and relaxation. This is very different from the drive to binge drinking, to get intoxicated. Whereas the euphoric effects of alcohol have been well studied, much less is known about factors driving to alcohol abuse. We have decided to assess the actions of alcohol at the molecular level, using sensitive methods and fluorescent molecular markers. A focus is naltrexone (NTX) which is approved by the Food and Drug Administration (FDA) for the treatment of alcohol use disorder (AUD). Recently, NTX has been advocated for the treatment of alcohol misuse as “one of the most underutilized interventions in medicine” [1].

Early experimental studies reporting the potential therapeutic effects of NTX in alcohol dependence [2, 3] were followed by clinical studies in human subjects. For example, NTX was found to reduce the feeling of “high” induced by alcohol in alcohol-dependent individuals [4]. However, the response was not universal and it has been suggested that differences in response may be genetically determined. The potential therapeutic effects of NTX for the treatment of AUD eventually led to an FDA approval for this indication, which is significant since very few medications for this condition are available. NTX was initially assumed to counteract alcohol-induced release of endogenous opioids acting on the MOP [5, 6].

It is commonly assumed that AUD is a consequence of the euphoriant activity of alcohol (positive reinforcement) and of craving, the urge to resume consumption in abstinence (negative reinforcement). It has also been proposed that positive reinforcement is mainly exerted via MOP and negative reinforcement is mainly exerted via kappa-opioid receptors (KOP) [7]. Although this model may seem rational for therapeutic developments, there are principal difficulties. In classic binding analysis, NTX is assumed to primarily act on MOP and has lower affinity and activity for KOP. This has been an opening for the clinical use of compounds with overlapping affinities for MOP and KOP such as pentazocine or buprenorphine. Our studies show that NTX has a significant influence on alcohol interactions with KOP – an activity that may be particularly relevant in binge drinking. In a series of publications using sensitive imaging technologies, we have observed that alcohol (ethanol, EtOH) in pharmacologically relevant concentrations directly affects glycosylphosphatidylinositol-enriched membrane domains, MOP, and KOP; these effects were largely blocked by NTX [8, 9]. A behavioral/neurochemistry analysis in mice observed a KOP supersensitivity and a hypodynamic side of the nucleus accumbens [10].

A valuable asset for drug development is the reported structural characterization of the dynorphin/KOP system. The X-ray structure of KOP with the antagonist JDTic was one of the first in the opioid receptor family [11]. The dynamics of the interaction between dynorphin and KOP was followed using nuclear magnetic resonance (NMR) [12]. Membrane lipids have been seen as a catalyst for dynorphin – KOP interaction: membrane attraction results in lower energy needed for the ligand to bind to the receptor [13]. These observations are probably also relevant for the NTX-KOP interaction and may be sensitive to the presence of alcohol. To probe the receptor binding sites, fluorescent NTX has been used previously [14]. Here we used a newly designed fluorescent NTX derivative (fNTX) and a fluorescent protein conjugate of KOP to assess the ethanol effects on KOP and KOP-NTX.

## MATERIALS AND METHODS

### Chemical reagents

Ethanol (EtOH, purity ≥ 99.5%) and NTX were purchased from VWR and Tocris. Methyl-β-cyclodextrin (mβCD) and Nalfulrafine (NFF) were purchased from Sigma-Aldrich. The less active opioid enantiomer of NTX, (+)-NTX was kindly provided by Dr. Kenner C. Rice [15]. The fluorescent NTX derivative (fNTX) with Alexa Fluor 633 was synthesized as detailed in the Supplement Information (fig. S15). The KOP selective antagonist, LY2444296 was supplied by Eli Lilly. All chemical compounds except for EtOH and (+)-NTX was suspended in dimethyl-sulfoxide (DMSO). (+)-NTX was suspended with MQ water. 1,2-dioleoyl-sn-glycero-3-phosphoethanolamine (DOPE) was conjugated with Abberior Star Red with a polyethylene glycol (PEG) linker (ASR-DOPE) [16]. MemGlow Nail Red 12S (NR12S) for lipid fluidity studies was purchased from Cytoskeleton, Inc.

### Cell culture

PC12 cells (American Type Culture Collection) were maintained in a humidified atmosphere containing 5% CO_2_ at 37°C in RPMI1640 medium (Gibco) supplemented with 10% horse serum (Gibco), 5% fetal bovine serum (Gibco) and 1% penicillin-streptomycin (10,000 U/mL, Gibco). We also transformed PC12 cells with a plasmid DNA encoding human KOP tagged with enhanced green fluorescent protein (PC12/hKOP-eGFP) as described previously [17]. For fluorescence measurements, PC12 cells were seeded in Lab-Tek 8-well chambered cover glass (Thermo Fisher Scientific) with 4.0 ×10^4^ cells/well.

For FLIM/FRAP measurements, PC12 cells were pre-treated at 37°C with antagonists (NTX, (+)-NTX for 30 min; LY2444296 for 15 min) or an agonist (NFF for 30 min), then treated with EtOH + antagonists/agonist for 1 h. The EtOH alone cells were pretreated with vehicle for 30 min, then treated with EtOH for 1 h. For cholesterol depletion, cells were treated for 3 h with 2.5 mM mβCD in serum free medium at 37°C [18]. Antagonists, agonist, mβCD and EtOH were diluted with FluoroBrite RPMI1640 (Gibco).

For dual-color Fluorescence Correlation Spectroscopy (dcFCS) of lipid fluidity and KOP dynamics, PC12 cells were treated with antagonists/EtOH as described above. Cells were further stained with ASR-DOPE for 5 min. To obtain eGFP brightness, untransfected PC12 cells were transfected with 100 ng of plasmid encoding eGFP, peGFP-N1 with 0.2 μL of lipofectamine 2000 (Thermo Fisher Scientific). After the transfection, PC12/eGFP cells were cultured for 24 h.

For Ca^2+^ imaging, PC12 cells were stained with 10 μM Fura Red in non-serum FluoroBrite RPMI1640 with 0.1% Pluronic F-127 (Invitrogen) for 3 h. Antagonists and EtOH were diluted with Dulbecco’s Phosphate Buffered Saline supplemented with 2.2 mM CaCl_2_, and 3.5 mM KCl. PC12 cells were treated with antagonists/EtOH as described above.

### Microscopic techniques and their data analysis

Detailed descriptions of microscopy techniques and data analysis form a separate chapter in the Supplementary Information.

## RESULTS

### Impact of ethanol on the KOP-surrounding environment

**Figure 1:**
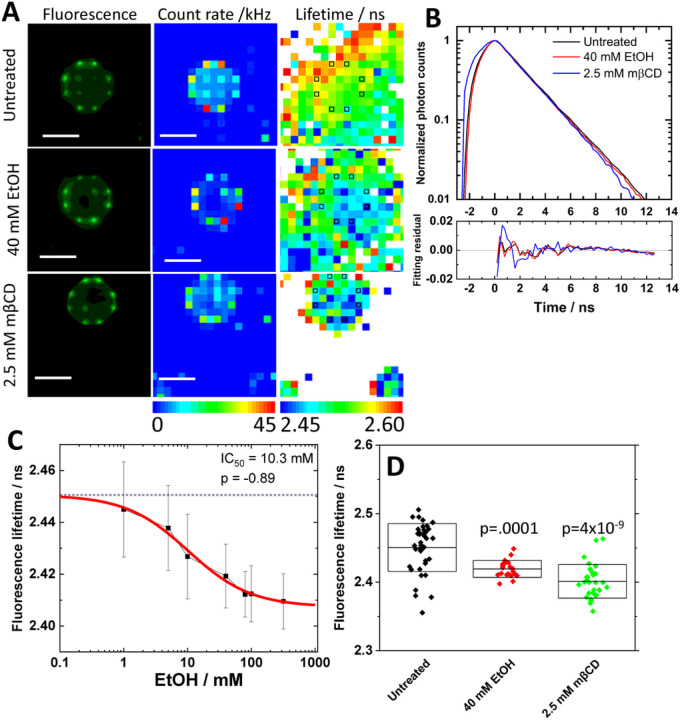
Impact of ethanol on the surrounding environment assessed with fluorescence lifetime (FL) analysis. **(A)** Fluorescence images/photon counts map taken with CMOS camera (left column) and spc3 SPAD camera (middle column). FL map (right column) generated by fitting analysis on FLIM curves shown in (B). Black squares indicate plasma membrane positions assigned by photon count map. Scale bar: 10 μm. **(B)** FLIM curves and fit residuals. Black: untreated, Red: 40 mM EtOH, Blue: 2.5 mM mβCD. **(C)** Dose response of FL against EtOH concentration. Best fit of dose-response curve determined 10.3 mM and −0.89 as IC_50_ value and allosteric factor (p), respectively. **(D)** Average ± Standard deviation of FL under the treatment with 40 mM EtOH and 2.5 mM mβCD. Statistical analysis was performed by two-tailed student’s t-test against untreated.

Ethanol is known to affect membrane lipid structures [8, 19, 20]. The size of nano-scale clusters and receptor density of KOP in the plasma membrane are also affected under EtOH treatment [9]. These studies indicate that EtOH may change the environment surrounding KOP and receptor self-organization in the plasma membrane. To further characterize the receptor environment, Fluorescence Lifetime Imaging Microscopy (FLIM) was carried out in PC12 cells expressing KOP-eGFP using our in-house instrument [21]. KOP-eGFP fluorescence was localized in the plasma membrane with and without the treatment of 40 mM EtOH (Fig. 1A, top and middle rows), suggesting EtOH treatment does not induce receptor internalization as observed with agonist (fig. S2A). FLIM curves in the plasma membrane, showed a small change (Fig. 1B). Fluorescence Lifetime (FL) was determined in several positions in the plasma membrane. A spatial FL map indicated shorter FL in the plasma membrane (Fig. 1A, right). This change in FL depended on the EtOH concentration with an IC_50_ value, 10.3 mM (Fig. 1C). Since dynamic properties and nano-scale clusters of KOP-eGFP are influenced by cholesterol-enriched membrane domains [18], FLIM measurement was performed in the plasma membrane under the cholesterol depletion by 2.5 mM mβCD. FL was significantly decreased (Fig. 1D). This suggests that EtOH affects dose-dependently the receptor-surrounding membrane environment including a change in the cholesterol-enriched membrane domain.

### Specificity of NTX binding to the KOP receptor and influence of EtOH

We have previously observed that NTX enhances formation of a larger nano-scale cluster of KOP [9]. To confirm the NTX effect on KOP in the plasma membrane, FLIM was also performed under the treatment with NTX or NTX + EtOH. NTX decreased FL in a dose-dependent manner, with a IC_50_ value 7.6 nM (Fig. 2A). To confirm that NTX binding triggered a change in the FL, we also tested two related compounds; 1) the inactive optical isomer, (+)-NTX [22] and 2) the KOP-selective agonist, nalfurafine (NFF) [23]. Each compound caused changes in FL, with the IC_50_ value 19 μM with (+)-NTX and 0.15 nM with NFF (fig. S1A and S2B). These values coincide with the binding affinity of these compounds to the KOP receptor estimated in other studies [22, 24, 25] (Fig. 2C, black). Interestingly, the dose-response curve with NTX in presence of 40 mM EtOH was shifted to a higher concentration (Fig. 2B), whereas dose dependency disappeared in the treatment with (+)-NTX and 40 mM EtOH (fig. S1B and S1C). On the other hand, 40 mM EtOH did not change the IC_50_ value of NFF (fig. S2C).

To further address whether the higher IC_50_ value of FL is caused by the lower binding affinity of NTX in the presence of 40 mM EtOH, a fluorescently labeled NTX (fNTX) was synthesized and tested in PC12 cells (fig. S3A). fNTX binding was reduced under the treatment with 40 mM EtOH, and was blocked by a large excess of non-labeled NTX and the KOP-selective antagonist, LY2444296 (fig. S3A, B), suggesting KOP-specific binding of fNTX in the plasma membrane. Dose-response curves of fluorescence intensity ratio (fNTX/KOP-eGFP) were determined to be 15.4 nM (fNTX) and 93 nM (fNTX + 40 mM EtOH) as EC_50_ values (Fig. 2D and 2E). NTX binding affinity to KOP was lower under the 40 mM EtOH, in agreement with our FLIM data (Fig. 2A–C). Fluorescence Recovery After Photobleaching (FRAP) analysis demonstrated that the immobile fraction of KOP-eGFP was the same with fNTX and NTX (Fig. 2F), suggesting that fNTX retained similar functionality.

### NTX reduces EtOH effect on lipid dynamics

**Figure 3:**
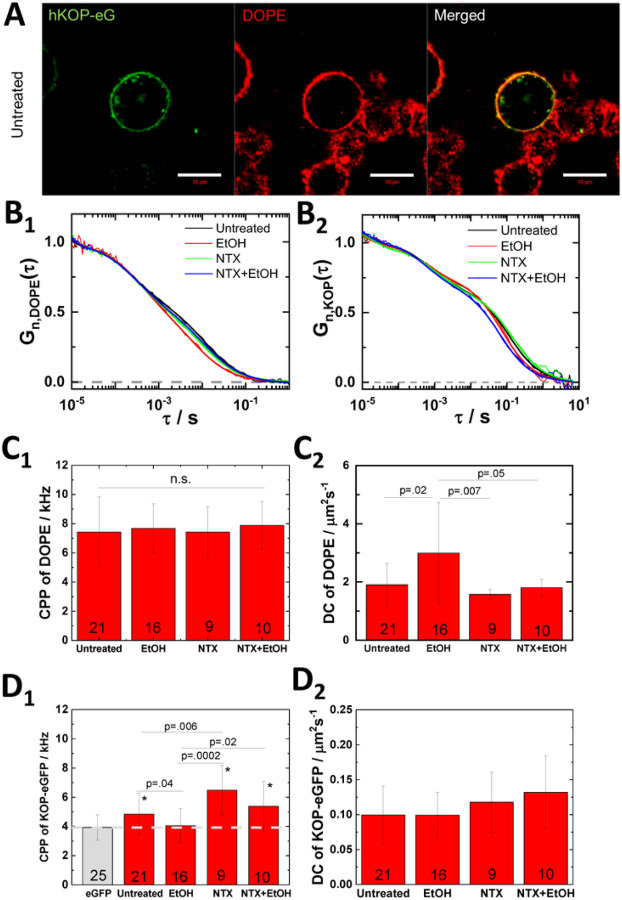
NTX reduces EtOH effect on lipid dynamics and KOP oligomerization. **(A)** Fluorescence images of untreated PC12 cells expressing KOP-eGFP (KOP-eG, green) and staining with ASR-DOPE (DOPE, red). Scale bar: 10 μm. **(B)** Autocorrelation curves of ASR-DOPE (B_1_) and KOP-eGFP (B_2_) in the plasma membrane under the treatments. **(C)** Fitting results from ASR-DOPE. Counts per particle (CPP) (C_1_) and diffusion coefficient (DC) (C_2_) of membrane-bound component of ASR-DOPE. **(D)** Fitting results from KOP-eGFP and eGFP (grey). CPP (D_1_) and DC of slow component (D_2_) of KOP-eGFP. eGFP was used as brightness standard for the monomeric form of KOP-eGFP. Number of measured single cells is shown at the bottom of bars in (C) and (D). Statistical analysis was performed by two-tailed student’s t-test in (C) and (D). *p<0.01 against eGFP.

To investigate the EtOH effect on lipid and receptor dynamics, we stained PC12 cells expressing KOP-eGFP with a lipid marker Abberior Star Red-labeled DOPE (ASR-DOPE) which clearly stained the plasma membrane and colocalized with KOP-eGFP in control cells (Fig. 3A) and EtOH-treated cells (fig. S4). Dual-color Fluorescence Correlation Spectroscopy (dcFCS) was carried out in the plasma membrane. Autocorrelation curves recorded with ASR-DOPE (Fig. 3B_1_) showed two distinct decay curves corresponding to diffusions of ASR-DOPE in the medium and in the plasma membrane, respectively. The counts per particle of ASR-DOPE (CPP; particle brightness in FCS) was not significantly changed under any condition (Fig. 3C_1_), whereas diffusion in the plasma membrane was significantly faster in the presence of 40 mM EtOH (Fig. 3C_2_). Pretreatment with 200 nM NTX significantly reduced the EtOH effect (Fig. 3C_2_). To address the NTX effect on EtOH-modulated lipid dynamics, we also tested (+)-NTX and the selective KOP antagonist, LY2444296 (LY) [26, 27]. As expected from FL measurement (fig. S1A), 200 nM (+)-NTX did not suppress the EtOH effect on lipid dynamics (fig. S5C). To study whether this is an NTX-specific or general effect of KOP antagonists, PC12 cells were treated with 100 nM LY2444296. There was no suppression of the EtOH effect (fig. S6B). This suggests that NTX modulates specifically lipid dynamics in a KOP-unrelated pathway. ASR-DOPE diffusion (fig. S7) and membrane fluidity using General Polarization (GP) analysis (fig. S8) in untransfected PC12 cells supported the EtOH and NTX effect on lipid dynamics. These results indicate that EtOH enhances lipid fluidity and that NTX reduces the EtOH effect in a KOP-independent manner.

### Effects of EtOH and NTX on KOP dynamics and dimerization

Receptor homodimerization has key roles for receptor functionality [28–30]. We computed counts per particle (CPP) of KOP-eGFP for analysis of its homodimerization. Recorded in untreated cells, CPP was significantly higher than eGFP (Fig. 3D_1_), suggesting that KOP is partially homodimerized. EtOH significantly reduced the CPP of KOP-eGFP to the level of eGFP, suggesting that KOP homodimers dissociate to the monomeric state. On the other hand, NTX enhanced KOP homodimerization and suppressed the EtOH effect during combined treatment. This effect was also observed under the treatment with 100 μM (+)-NTX, but not with 200 nM (+)-NTX (fig. S5D). The selective KOP-antagonist, LY2444296 did not interfere with EtOH-induced dissociation of KOP dimer (fig. S6C). We suggest that EtOH and NTX modulate nano-scale cluster formation and number of KOP in the nano-scale cluster, affecting KOP homodimerization.

Our previous data indicate that KOP is non-randomly organized into nano-domains [18]. We also performed total internal reflection microscopy-based FCS (TIR-FCS) to determine the KOP-eGFP confinement in membrane domains by diffusion law analysis (fig. S9D). The intercept of linear regression under 40 mM EtOH was significantly reduced, suggesting that EtOH reduces KOP-eGFP confined to the membrane domains. Diffusion coefficient (DC) was insensitive under each treatment in PC12 cells, whereas significant difference in U2OS cells (Fig. 3D_2_ and fig. S9C). FRAP analysis addressed a reduction of the immobile fraction of KOP-eGFP with EtOH and an increase of that fraction under the treatment with NTX and NTX + EtOH (fig. S10). This may suggest that membrane domain localization of KOP-eGFP contributes to generate immobile receptor in the plasma membrane and EtOH redistributes KOP-eGFP to mobile fraction.

### EtOH and NTX modulate calcium signaling via KOP-dependent and independent pathways

**Figure 4:**
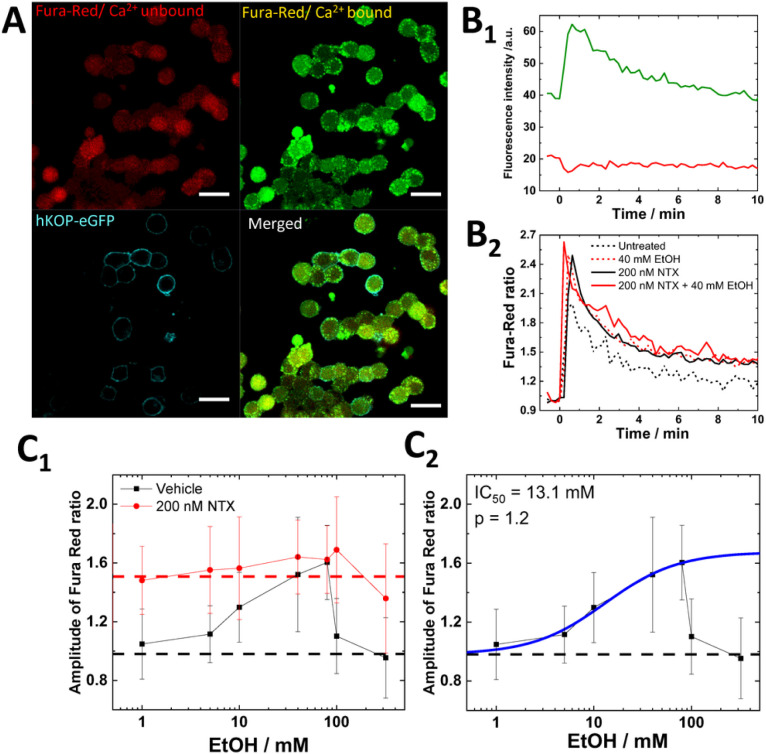
EtOH and NTX modulate calcium signaling via KOP-dependent and -independent pathways. Fluorescence images of untreated PC12 cells expressing KOP-eGFP and staining with Fura Red. Red: Ca^2+^-unbound Fura Red, Green: Ca^2+^-bound Fura Red, Cyan: KOP-eGFP. Scale bar: 20 μm. **(B**_**1**_**)** Time-series of fluorescent intensity of Fura Red during K^+^ depolarization. Green: Ca^2+^-bound Fura Red, Red: Ca^2+^-unbound Fura Red. **(B**_**2**_**)** Fura Red ratio normalized before the K^+^ stimulation. Black dashed line: Untreated, Red dashed line: 40 mM EtOH, Black solid line: 200 nM NTX, Red solid line: 200 nM NTX and 40 mM EtOH. **(C**_**1**_**)** Dose-dependency of amplitude of Fura Red ratio. Black: vehicle with EtOH, Red: 200 nM NTX with EtOH. Dashed line: mean value under the vehicle without EtOH (black) and with the 200 nM NTX alone (red). **(C**_**2**_**)** Best fit of dose-response curve (blue) to the data with EtOH gave 13.1 mM and 1.2 as IC_50_ and allosteric factor (p), respectively.

To address the impact of lipid dynamics modulated by EtOH and NTX for the function of KOP, we performed Ca^2+^ imaging in KOP-transfected cells with Fura Red (Fig. 4A). After K^+^ depolarization, fluorescence intensity of Ca^2+^-bound Fura Red dramatically increased while fluorescence intensity of Ca^2+^-unbound Fura Red was decreased (Fig. 4B_1_). The Fura Red ratio was calculated as the ratio of Ca^2+^-bound intensity and Ca^2+^-unbound intensity (Fig. 4B_2_). EtOH-treated KOP cells showed dose-dependent increment of the Fura Red ratio up to 80 mM EtOH, followed by a sudden drop to untreated levels with over 100 mM EtOH (Fig. 4C_1_). The IC_50_ value was determined to be 13.1 mM EtOH (Fig. 4C_2_), in good agreement with the IC_50_ value in FL (10.3 mM) (Fig. 1C). Cholesterol depletion caused similar changes of the Fura Red ratio as EtOH treatment (fig. S11), suggesting that the EtOH modulated Ca^2+^ influx *via* the modulation of the environment of KOP-eGFP including deformation of cholesterol-enriched membrane domains. To clarify whether this was a KOP-mediated pathway or not, Ca^2+^ imaging was performed in untransfected PC12 cells, showing a higher Fura Red ratio in untreated (black dashed line) compared with EtOH-untreated KOP-expressing cells (Fig. 4C_1_) and a gradual decrease of Fura Red ratio (fig. S12). This is in good agreement with previous studies that a L-type channel is inhibited and a non-L-type channel is partially inhibited by EtOH [31]. This suggests that KOP inhibits Ca^2+^ signaling at a basal level and that the enhancement of Ca^2+^ influx with EtOH treatment is KOP-mediated. To clarify the impact of NTX on Ca^2+^ influx, we pre-treated KOP-expressing cells and untransfected cells with 200 nM NTX. EtOH-untreated KOP-expressing cells showed higher Fura Red ratio and a constant Fura Red ratio even under the over 100 mM EtOH conditions (Fig. 4C_1_). The constant Fura Red ratio was also confirmed in untransfected cells (fig. S12). These data suggest that NTX affects live cells in two aspects; 1) direct binding to KOP in the plasma membrane and 2) KOP-unmediated modulation of cholesterol-enriched membrane domains which is in good agreement with our FCS data (Fig. 3). To further characterize the two effects of NTX on Ca^2+^ influx, we tested (+)-NTX (fig. S13) and LY2444296 (fig. S14). 200 nM (+)-NTX clearly showed no effect on Ca^2+^ influx (fig. S13, red), while 100 μM (+)-NTX slightly increased Ca^2+^ uptake without EtOH treatment, following similar changes under the co-treatment with EtOH (fig. S13, blue). LY2444296 (100 nM) was only found to enhance Ca^2+^ influx as a direct KOP antagonistic effect (Fig S14), via direct binding to KOP, but no KOP-unmediated effect (Fig. S6). Altogether FL data (Figs. 1 and 2) and FCS data (Fig. 3), suggest that EtOH modulates Ca^2+^ influx via changes of membrane environment, in particular deformation of cholesterol-enriched membrane domains. NTX does not only show the KOP-mediated antagonistic effects on Ca^2+^ influx, but also KOP-unmediated effects linking lipid dynamics and the receptor surrounding environment.

## DISCUSSION

Alcohol abuse and dependence remain the most significant substance abuse problems worldwide and in the US alone, more than 140,000 people are dying from alcohol-related causes annually [32]. There is a choice whether psychotherapy or medication have superiority for treatment, the majority of cases with AUD receive no treatment at all.

Earlier studies of medication in AUD have been subjected to a meta-analysis, showing the NTX and acamprosate are superior to placebo; NTX is particularly effective in heavy drinking and prevention of recurrence [33]. A more recent survey supports the efficacy of NTX. Moreover, effectiveness of extended-release NTX medication [34] was recently demonstrated. However, NTX-based prescriptions were primarily given to higher income males with private insurance, leaving women and minorities without such intervention [35]. A certain rise in AUD have been recorded during the covid-19 pandemic [36], prompting an editorial advocating higher rate of NTX prescriptions [1].

Behavioral effects mediated by KOP differ markedly from those of the other opioid receptors, MOP and the delta-opioid receptor (DOP). Kappa-agonists are not self-injected and clinical use is compromised by psychotomimetic side effects. It has even been proposed that the overt euphorigenic effects of MOP and DOP pathways are related to positive reinforcement whereas effects on KOP are balancing and related to the negative reinforcement (craving) [37]; both effects have been related to AUD.

NTX has found a therapeutic niche for the treatment of AUD and is one of the very few medications that can be prescribed for this indication. One characteristic of AUD in humans is that dependent subjects will consume alcohol to relieve or avoid withdrawal symptoms. Similarly, in preclinical studies, alcohol postdependent rats exhibit an alcohol dependence syndrome that is characterized by both somatic and motivational withdrawal symptoms that usually begin after 6 to 8 hours of abstinence and engage in excessive drinking when alcohol is made available again. Using a rat model of alcohol dependence (i.e, chronic intermittent alcohol vapor exposure) we showed that NTX decreased alcohol intake in nondependent rats, regardless of sex and abstinence time point [38]. In postdependent rats, NTX significantly decreased the exaggerated alcohol intake only at a delayed abstinence time point (i.e., 6 weeks) in males, whereas it similarly reduced alcohol drinking in females at 8 h, 2 weeks, and 6 weeks abstinence time points. These findings further support targeting the endogenous opioid system to prevent excessive drinking that is characteristic of AUD, even after long periods of abstinence and further suggest that alcohol dependence causes neuroadaptations [38].

The access of a fluorescent derivative of NTX was a priority in the study. While fluorescent NTX derivatives have been described before [14], the strategy here was to extend the separation of the fluorescent marker to NTX by a longer linker, considering the X-ray analysis data showing that the JDTic binds in a deep pocket [11] and NMR analysis of the KOP/dynorphin interaction [12].

NTX has been reported to have lower affinity for KOP as compared to MOP and may therefore not be considered a KOP antagonist. However, under our conditions, affinity is strong and more in line with previous studies using competition assays in transfected cell cultures that identified approximately equal affinity of NTX for KOP and MOP [39]. Constitutive activity and inverse agonism was also observed, which increased after agonist pretreatment [39]. To approach the effects of alcohol on both MOP and KOP at a molecular level, we introduced high-resolution molecular imaging with FCS to follow the dynamics in cell culture of MOP and KOP labeled with fluorescent tags. The addition of pharmacologically relevant concentrations of EtOH influenced their lateral movements in the plasma membrane [40]. Significantly, EtOH-induced effects showed differences between MOP and KOP, with higher presence of MOP in the membrane, whereas KOP presence declined. Differences related to EtOH-induced effects were also observed with super-resolution microscopy [8, 9].

In our studies, we have also used high-resolution technologies to investigate the effects of EtOH on both MOP and KOP. As expected, NTX blocks the activation of MOP. In cell culture at the ultrastructure level, EtOH affects the distribution of both MOP and KOP (induces the formation of smaller and less occupied MOP and KOP nanodomains) [9]. These studies also revealed that NTX induces formation of larger and more occupied KOP nanodomains and that NTX pretreatment has protective effects against EtOH-induced changes in nano-organization of both receptors [9].

Another approach to the specificity of the studies effects is the use of stereoisomers. The (+) isomer of NTX ((+)-NTX) was available to us. This isomer has its own pharmacologic profile and shows equipotent binding for MOP and the toll-like receptor TR4, and interacts with opioid (morphine) analgesia [41, 42] as well as drug reward [43] The results are clear, the (+) isomer is much less active demonstrating that the NTX effects we observe are mediated by interaction with KOP and not due to off-target effects (Fig. 2).

**Figure 5:**
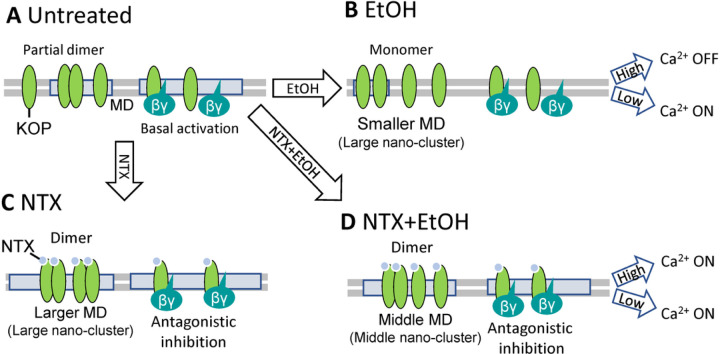
Modeling interaction of NTX, ethanol (EtOH) and the kappa opioid receptor (KOP). **(A)** KOP diffuses freely and partially localizes in the cholesterol-enriched membrane domains (MDs) and forms partially homodimers. MD-localized KOP is constitutively active and inhibits a Ca^2+^ channel. **(B)** Under treatment with EtOH, KOP dissociates to monomers in the fluidic plasma membrane. The Ca^2+^ channel is ON-state under lower EtOH concentration (0 mM – 80 mM). The Ca^2+^ channel may be effectively inhibited in MDs. Under high EtOH concentration (> 100 mM), Ca^2+^ channel may be inhibited generally. **(C)** Under treatment with NTX, larger nano-scale KOP clusters are formed*. NTX-liganded KOP forms preferentially homodimers in the larger MDs. Ca^2+^ channel is released from constitutive inhibition of KOP by NTX. **(D)** EtOH-modulated lipid dynamics is suppressed by NTX. Middle-sized of nano-scale clusters are formed. NTX also plays a role of the antagonist at KOP, thus the Ca^2+^ channel is released by KOP inhibition. * Model based on present data and superresolution analysis [9].

Based on this work, we have developed a model of direct/indirect actions of NTX to KOP (Fig. 5). In untreated condition, KOP has constitutive activity. It is partly in a homodimer form and causes Ca^2+^ channel inhibition. EtOH treatment dissociates KOP to monomers and induces deformation of cholesterol-enriched membrane domains. Since the Ca^2+^ channel may be effectively inhibited by KOP in the cholesterol-enriched membrane domains, Ca^2+^ influx is enhanced under the lower EtOH condition (1 ~ 80 mM). On the other hand, higher EtOH concentration (> 100 mM) distorts the membrane structure and induces Ca^2+^ channel inhibition.

Under the NTX condition, KOP forms larger nano-clusters and KOP is preferably present as homodimers. NTX also shows antagonistic effect on KOP, following the enhancement of Ca^2+^ influx. These NTX effects are sustained even with EtOH. Both NTX and EtOH affect the cholesterol-enriched membrane domains (KOP nano-clusters) as observed in previous study [9]. With EtOH cholesterol-enriched membrane domains of intermediate size are formed, and KOP is preferentially in homodimers even though to a lesser extent than with NTX alone. The Ca^2+^ influx remains the same in the whole EtOH concentration range, and lipid dynamics does not change. The EtOH-induced enhancement of Ca^2+^ influx was suppressed by NTX. Also, EtOH-induced distortion of Ca^2+^ influx under higher EtOH concentration was inhibited by NTX.

For comparison, we also included a known KOP antagonist a homologue of JNJ-67953964, LY2444296, and a NTX-related agent, nalfurafine, a KOP agonist recently introduced in the treatment of itch (as developed in patients receiving opiates chronically) [44]. We confirm that nalfurafine induces KOP internalization as a KOP agonist and like the natural ligand dynorphin. The observed activity of the LY2444296 confirmed KOP antagonism and as is shown here, NTX and LY2444296 share binding sites.

It can be noted that KOP has been identified as one of the strongest genetic linkages in major depressive disorder along with the D2R (dopamine receptor 2) [45]. It is noticeable that there is a striatonigral dynorphin pathway reciprocal to the classic nigrostriatal dopamine pathway [46, 47]. The close connection between two potentially relevant neurotransmitter systems may be an indication of a functional relationship. A recent “fast-fail” study of JNJ-67953964 (a.k.a. CERC-501 and LY2456302) in major depressive disorder showed activity in anhedonia [48]. It is now in Phase III trial as Aticaprant. Another chemically distinct KOP antagonist BTRX-335140, Navacaprant is in Phase II clinical trial in depression [49]. To our knowledge, there are so far no clinical studies of KOP antagonists in AUD.

### Concluding remarks

NTX is an analog of naloxone – a well-known opiate antidote in emergencies. NTX was developed as a long-acting MOP antagonist to protect against further intoxication. Its activity in AUD has been hard to conceptualize. As shown here, NTX has potent activity on KOP as an inverse agonist and takes actions on MOP. These receptors are structurally related. However, the neuronal pathways with both receptors are functionally highly different and there is no reason to see them as connected. A formal model with alcohol activating MOP (and euphoria) has been linked to release of endogenous opioids (enkephalins). The KOP system is seen as the origin of craving (and release of dynorphin peptides). Our model suggests that alcohol acts directly on KOP and that NTX acts as an inverse agonist blocking constitutive and alcohol-induced activity. Binge drinking (to intoxication) which is of medical concern is very different from social alcohol use [50].

The interactions of alcohol and NTX and the opioid receptors, particularly MOP, have been studied with a variety of technologies [6, 51]. The current data illustrates that the KOP receptor is also a relevant target.

## Figures and Tables

**Figure 2:** Effect of EtOH on NTX interaction with the KOP receptor. **(A, B)** Dose-response of FL under NTX (A) and NTX with 40 mM EtOH (B). Best fit of dose-response curve determined 7.6 nM and 1.0 (A) and 730 nM and −0.66 (B) as IC_50_ and allosteric factor (p), respectively. **(C)** Relationship of IC_50_ value in FL with binding affinity of agonist/antagonist to KOP; Nalfurafine (NFF), naltrexone (NTX) and (+)-NTX, respectively. Reported binding affinity was referred from [22, 24, 25]. Binding affinity of (+)-NTX to KOP was estimated from binding affinity of (+)-NTX to MOP and ratio of binding affinity of NTX. Black: Vehicle, Red: 40 mM EtOH. **(D, E)** Dose-dependency of fluorescence intensity ratio in the plasm membrane (fNTX/KOP-eGFP) in the absence **(D)** and presence **(E)** of 40 mM EtOH. Best fit of dose-response curves determined EC_50_ and allosteric factor (p). **(F)** Immobile fraction of KOP-eGFP in the plasma membrane assessed by FRAP. Statistical analysis was performed by two-tailed student’s t-test.

## Data Availability

Raw data can be provided for investigations from S.O.
